# A comparative analysis of large language models for providing oral cavity cancer information

**DOI:** 10.1038/s41598-026-53630-0

**Published:** 2026-05-23

**Authors:** Eda İzgi, Turan Canmurat İzgi, Ceren Mordağ Çiçek

**Affiliations:** 1 Department of Oral and Maxillofacial Surgery, Gülsüm Güral Faculty of Dentistry, Kütahya Health Science University, Inköy District, Eskisehir Highway Blvd. No: 65, Center, Kütahya, Turkey; 2 Department of Otorhinolaryngology, Faculty of Medicine, Kütahya Health Sciences University, Kütahya, Turkey; 3 Department of Medical Oncology, Faculty of Medicine, Pamukkale University, Denizli, Turkey

**Keywords:** Artificial intelligence, Health literacy, Quality of health care, Large language models, Mouth neoplasms, Cancer, Health care, Medical research, Oncology

## Abstract

This study aimed to comparatively evaluate the medical information delivery capacity and content quality of current large language models (LLMs), specifically ChatGPT (GPT-5.2), Gemini (3.1), and DeepSeek (V4), regarding oral cavity cancer (OCC) based on expert opinions. 20 open-ended questions addressing the risk factors, diagnosis, and treatment of OCC were directed to the three models. The responses were evaluated using a blinded method by 31 expert physicians from Oral and Maxillofacial Surgery, Otorhinolaryngology (ENT), and Medical Oncology. The Modified Global Quality Scale (1–5 points) was utilised for evaluation. Statistical analyses were performed using Kruskal–Wallis, ANOVA, and Bonferroni post-hoc tests, while Fleiss’ Kappa coefficient determined inter-expert consistency. The general performance scores of the models were high (3.57–4.15). In the overall assessment, Gemini received statistically significantly higher scores than the DeepSeek model (*p* = 0.036). Significant performance differences were identified across 15 of 20 questions (*p* < 0.05); ChatGPT excelled on clinical and treatment-oriented questions, while Gemini stood out on comprehensive informational items. While no statistically significant difference was found among the specialist groups for the overall evaluation and 19 out of 20 questions (*p* > 0.05), a significant difference was observed solely for Q6 (*p* = 0.042). Although LLMs have the potential to generate high-quality information about OCC, their performance varies by content type and model architecture. While Gemini demonstrated more consistent performance overall, expert supervision remains essential before these tools can be used as reliable sources of clinical information. Clinicians must be aware of the specific strengths and limitations of different LLMs in OCC to better guide patients who increasingly use such tools for medical information.

## Introduction

Artificial Intelligence-based Large Language Models (LLMs) are technologies that enable the development of computer systems capable of decision-making through learning and analytical capabilities during the pursuit of predetermined objectives^1^. The evolution of LLMs and their associated methodologies is regarded as one of the revolutionary transformations of the modern era. The proficiency of LLMs across various application domains is expanding rapidly, with their integration into the medical field standing at the forefront of these technological advancements^2^. Recently, patients have increasingly turned to the internet as a primary resource for accessing health-related information^3^. While the use of the internet for health information-seeking is not new, the emergence of advanced LLMs has introduced significant shifts in this landscape. These tools facilitate more personalized and interactive approaches to diagnosis, screening, and treatment planning. Nevertheless, the overall quality of information provided by these platforms, necessitates close scrutiny, particularly concerning critical conditions such as OCC, where early diagnosis is of paramount importance^4^.

OCCs represent increasingly significant global health concerns. Annually, approximately 377,000 cases of OCC and 98,000 cases of oropharyngeal cancer are diagnosed worldwide, accounting for roughly 178,000 and 48,000 deaths, respectively^5^. OCC constitutes a severe health challenge characterized by high mortality rates, primarily since these malignancies are often diagnosed at advanced stages. The primary factors contributing to the delayed diagnosis of OCC include low socioeconomic status, advanced age, cognitive impairment, and anxiety. Furthermore, some patients delay seeking medical consultation even after noticing symptoms, as they frequently misinterpret these signs as benign conditions, such as traumatic or aphthous ulcers^3^. Early detection plays a pivotal role in determining the prognosis of OCC^6^.

In this context, the quality of information resources that can enhance early-stage awareness among individuals is of paramount importance. Consequently, a systematic evaluation of the performance of LLM-supported platforms in providing information in this field may yield valuable insights for both healthcare professionals and users. The objective of this study is to comparatively analyse the performance of three current LLMs (ChatGPT [GPT-5.2], Gemini [3.1], and DeepSeek [V4]) in providing patient information regarding OCC, based on evaluations conducted by multidisciplinary medical specialties (Oral and Maxillofacial Surgery [OMFS], Otorhinolaryngology [ENT], and Medical Oncology [MO]).

## Materials and methods

### Study design

In this study, structured to assess the capacity of LLMs to deliver health-related information, a total of twenty open-ended questions with identical content were directed to each model. The questions encompass general information, risk factors, early symptoms, screening methods, and treatment options for OCC.

### Sample size calculation

The sample size required to test the hypothesis of significant differences among the three LLMs (ChatGPT, Gemini, and DeepSeek), based on expert evaluations, was calculated using G*Power software (version 3.1.9.7; Heinrich-Heine-Universität Düsseldorf, Germany). Given that the same experts evaluated all three models, a repeated-measures ANOVA design (with a within-subjects factor) was used. In the absence of directly comparable prior studies, the effect size was estimated using Cohen’s recommendations, with a medium effect size assumed (f = 0.25). The calculation was performed using an alpha level of 0.05 and a statistical power of 0.80. Based on these parameters, the minimum required total sample size was determined to be 30 evaluations.

### Data collection

All LLMs were accessed between February 15 and February 28, 2026, via their respective official web interfaces using premium subscription accounts to ensure access to the models’ highest processing capacities, most advanced reasoning capabilities, and the most updated high-tier versions. To evaluate the models at their maximum technical capacity and ensure high data quality, all queries were directed to the LLMs in English, and the generated responses were analysed in the same language. Regarding the prompting protocol, each query was structured as a clear, direct, and identical command to maintain consistency across all platforms. To minimize potential personalization effects and ensure reproducibility, each of the 20 queries was initiated as a completely independent ‘New Chat’ session for every model. This protocol ensured that the models had no access to previous conversational contexts, thereby preventing any inter-query influence or cumulative bias (zero-shot prompting). Features such as custom instructions, personalized memory, or pre-existing conversational contexts were disabled. Furthermore, default system configurations were used for all models; technical parameters (such as API temperature or top-k settings) were not manually modified, as these are provider-optimized within the web interfaces to deliver the most stable, high-quality outputs for general users. The LLM-generated responses were subsequently copied, transferred to a digital document (Microsoft Word), and analysed in their original, unaltered forms. These standardized conditions were intentionally selected to simulate the typical experience of a patient seeking health information under real-world conditions, where technical parameters are generally not adjusted by the end user.

### Large language models (LLMs)

The LLMs utilised in this study are systems characterized by advanced natural language processing capabilities and are publicly accessible online. The information retrieved from these platforms is based on data current as of February 2026. It should be noted that the versions and capabilities of these LLMs are subject to periodic updates.

#### ChatGPT (OpenAI, San Francisco, CA; GPT-5.2 architecture)

ChatGPT, developed by OpenAI and built upon the GPT-5.2 architecture, is an advanced LLM. Launched in mid-2025, this iteration was trained on unprecedented-scale, high-quality text, visual, and agentic data. Beyond text-based interactions, it assists users in various cognitive tasks, including agentic task execution, complex reasoning, and advanced coding and analysis. For this study, ChatGPT was accessed via https://chat.openai.com/ (Version: ChatGPT-5.2, February 2026)^7^.

#### Gemini (Google DeepMind, Mountain View, CA; Version 3.1)

Gemini is an LLM-based system developed by Google DeepMind. Serving as the successor to the Bard platform, Gemini was introduced in late 2023 and is available for tasks such as text generation, data analysis, coding, and question answering. In this study, Gemini was accessed via https://gemini.google.com/ (Version: Gemini 3.1, February 2026)^8^.

#### DeepSeek Chat (DeepSeek AI, Hangzhou, China; Version V4)

DeepSeek Chat is an open-source LLM developed by DeepSeek AI in China. Released in 2024, the model was primarily trained on English and Chinese corpora and possesses the capacity for multimodal and multilingual content generation. DeepSeek Chat was accessed via https://chat.deepseek.com/ (Version: DeepSeek V4, February 2026)^9^.

### Demographic characteristics and attitudes toward LLMs

The 31 evaluating specialists were recruited using a convenience sampling method from three different tertiary healthcare centres. The demographic profiles and attitudes of these participants toward LLM-based medical information sources were evaluated, including data on gender, age range, medical specialty (OMFS, ENT, and MO), total years of professional experience, and employment sector. Furthermore, their usage frequency and trust levels regarding LLMs were recorded to interpret the potential influence of individual expert characteristics on the evaluation process.

To ensure a strictly blinded assessment, the LLM-generated responses were transferred to a standardized digital evaluation form created via the Google Forms platform. Each expert accessed the form via a unique link, in which the model identities were replaced with generic codes (e.g., Model A, Model B). This digital interface allowed for the randomized presentation of outputs and ensured that experts recorded their scores independently without seeing the assessments of other raters. All responses were collected anonymously and utilized solely for descriptive statistical analysis.

### Questions directed to the LLMs

To evaluate the clinical utility and quality of the LLMs, a standardized set of 20 open-ended questions was developed (Table 1). These questions were specifically curated to reflect clinical priority areas and patient concerns, categorized according to the National Comprehensive Cancer Network (NCCN) Guidelines for Oral Cancer and World Health Organization (WHO) oral health standards^10^.

**Table 1:** Questionnaire for assessing LLM outputs.

Question No	Questions Addressed
Q1	What are the symptoms of oral cavity cancers?
Q2	What are the risk factors for oral cavity cancers?
Q3	What is the relationship between HPV and oral cavity cancers?
Q4	What are the histopathological types of oral cavity cancers?
Q5	What are the most common sites for oral cavity cancers?
Q6	What are the distributions of oral cavity cancers based on age and gender?
Q7	According to epidemiological studies, in which populations do oral cancers occur most frequently?
Q8	What is the global awareness level of oral cavity cancers?
Q9	What tools are used in the diagnosis of oral cavity cancers?
Q10	What is the TNM staging system for oral cavity cancers?
Q11	What is the significance of depth of invasion in oral cavity cancers?
Q12	What are the neck metastasis rates of oral cavity cancers?
Q13	What are the distant metastasis rates of oral cavity cancers?
Q14	What is the recurrence rate in oral cavity cancers?
Q15	What are the survival rates for oral cavity cancers?
Q16	What are the treatment principles for oral cavity cancers?
Q17	What are the surgical treatment methods for oral cavity cancers?
Q18	What should be the approach to neck lymph nodes in oral cavity cancers?
Q19	What is the role of neoadjuvant and adjuvant therapy in oral cavity cancers?
Q20	What can be done to prevent oral cavity cancers?

LLM, Large Language Model; HPV, Human Papilloma Virus; TNM, Tumour, Node, Metastasis.

### Evaluation methodology

The responses generated by the three LLMs were evaluated for content by a panel of 31 specialist physicians. A total of 31 clinicians specializing in OMFS (n = 10), ENT (n = 10), and MO (n = 11) participated in the evaluation process. Prior to the formal scoring phase, a pilot calibration session was conducted with representative specialists (n = 3, one from each specialty) to standardise the interpretation of the evaluation criteria and minimize inter-observer variability. To eliminate cognitive bias, a double-blind protocol was implemented where neither the primary investigators nor the evaluating specialists were aware of the specific source of the generated text during the scoring phase.

The quality of the LLM responses was evaluated using the modified Global Quality Score (mGQS), a 5-point Likert scale^11^. During the assessment, the responses from each LLM were presented to the experts in an anonymous, randomized order, ensuring that the evaluators remained blinded to the models’ identities. The experts were asked to rate each response on a scale of 1 (poor quality) to 5 (excellent quality) using a holistic scoring rubric (Table 2).

**Table 2:** mGQS scoring criteria for assessing LLM outputs.

Score	Description
1	Poor quality, poor information flow, most information is missing, not useful at all for patients or education
2	Generally poor quality and flow; some information is present but significant gaps exist; limited use for patients or education
3	Moderate quality; reasonable flow but some important information is missing; somewhat useful for patients or education
4	Good quality and flow; most relevant information is covered; useful for patients or education
5	Excellent quality and flow; comprehensive and highly useful for patients or education

mGQS, Modified Global Quality Scale; LLM, Large Language Model.

This evaluation was guided by four fundamental pillars derived from the mGQS framework: the quality of the information, the flow of the information, the comprehensiveness of the content, and its utility for patient education^11^. In accordance with the mGQS methodology, these criteria were not scored independently as separate variables; instead, they served as a qualitative framework for the experts to assign a single global quality score ranging from 1 to 5. For instance, a score of 1 represents poor quality and information flow, with most information missing, whereas a score of 5 indicates excellent quality and comprehensive information that is highly useful for patients or education. This approach ensures that the structural integrity of the response and its practical value are captured within a single, representative metric for statistical analysis.

### Statistical analysis

Statistical analysis of the study data was performed using the Statistical Package for the Social Sciences (SPSS) software, version 27 for Windows (SPSS Inc., Chicago, IL, USA). Descriptive statistics are presented as frequencies, percentages, means, standard deviations (SD), minimums, maximums, and medians. The normality of the data distribution was assessed using the Shapiro–Wilk test, and the homogeneity of variances was evaluated with Levene’s test. To evaluate the inter-rater reliability of the categorical OCC information responses among multiple evaluators, Fleiss’ Kappa (κ) analysis was employed. The strength of agreement was interpreted as weak (0.01–0.20), moderate (0.21–0.40), moderate-good (0.41–0.60), good (0.61–0.80), or very good (0.81–1.00)^12^. Since the same experts evaluated all three large LLMs, model performance comparisons were primarily conducted using the Friedman test. In addition, for variables meeting parametric assumptions, repeated-measures Analysis of Variance (ANOVA) was applied. The assumption of sphericity was assessed using Mauchly’s test; if violated, the Greenhouse–Geisser correction was applied to adjust the degrees of freedom. When statistically significant differences were detected, post-hoc pairwise comparisons were performed using Bonferroni correction. For comparisons among independent specialty groups (OMFS, ENT, and MO), one-way Analysis of Variance (ANOVA) was used when parametric assumptions were met, whereas the Kruskal–Wallis test was applied when these assumptions were violated. When statistically significant differences were identified, post-hoc pairwise comparisons were conducted using Bonferroni-adjusted tests. For all statistical analyses, the Type I error rate (α) was set at 0.05.

## Results

The study included 31 specialist physicians, of whom 58.1% (n = 18) were male. All participants were aged 25–44 years (25–34: 45.2%, n = 14; 35–44: 54.8%, n = 17). Distribution across specialties was balanced, involving MO (35.5%, n = 11), OMFS (32.3%, n = 10), and ENT (32.3%, n = 10). Nearly half the cohort (48.4%, n = 15) possessed 6–10 years of professional experience, and 58.1% (n = 18) held academic appointments. Regarding LLM utilization, 38.7% (n = 12) reported occasional use (several times per week), while 19.4% (n = 6) had never used LLMs. Reported trust in LLMs was predominantly moderate to high, with the majority of scores concentrated at level 3 (51.6%, n = 16) and level 4 (41.9%, n = 13).

The inter-rater reliability analysis revealed significant agreement (*p* < 0.05) in 8 out of 20 questions (Q7–Q11, Q17, Q18, and Q20). The calculated κ values for these items indicated a range of weak-to-moderate consistency among raters. The highest levels of agreement were observed for Q7 (κ = 0.351, *p* < 0.001) and S20 (κ = 0.314, *p* = 0.002), both falling within the moderate agreement range. However, for the remaining 12 questions, no statistically significant consensus was reached, suggesting higher variability in rater responses for those specific items.

Performance scores for OCC information across the three LLM platforms were evaluated. Statistically significant differences between the groups were identified in 15 of the 20 questions (75%) (*p* < 0.05, Table 3), indicating that the quality and scope of medical information provided by the platforms were not uniform. In contrast, no significant differences in scores were observed for questions Q4, Q8, Q11, Q18, and Q20 (*p* > 0.05), where the platforms provided a comparable level of information.

**Table 3:** Comparison of scores assigned to three different LLM platforms for the addressed questions.

Question No	A		B		C		Test statistic	*p*	Post-hoc (Bonferroni)
	Median (Min–Max)	Mean ± SD	Median (Min–Max)	Mean ± SD	Median (Min–Max)	Mean ± SD			
Q1	3.00 (1.00–5.00)	3.16 ± 0.93	4.00 (2.00–5.00)	3.97 ± 0.80	5.00 (3.00–5.00)	4.55 ± 0.62	KW = 33.703	< 0.001*	A < B; A < C; B < C
Q2	3.00 (2.00–5.00)	3.39 ± 0.88	4.00 (3.00–5.00)	4.29 ± 0.64	5.00 (4.00–5.00)	4.55 ± 0.51	KW = 29.306	< 0.001*	A < B; A < C
Q3	3.00 (1.00–5.00)	3.23 ± 1.02	5.00 (3.00–5.00)	4.52 ± 0.72	4.00 (3.00–5.00)	4.19 ± 0.70	KW = 26.789	< 0.001*	A < B; A < C
Q4	4.00 (2.00–5.00)	3.81 ± 1.05	4.00 (2.00–5.00)	4.03 ± 0.91	4.00 (2.00–5.00)	3.71 ± 0.86	KW = 1.912	0.385	–
Q5	5.00 (2.00–5.00)	4.52 ± 0.77	4.00 (2.00–5.00)	3.77 ± 0.96	3.00 (1.00–5.00)	3.10 ± 1.22	KW = 23.830	< 0.001*	C < A; B < A
Q6	3.00 (2.00–5.00)	3.45 ± 0.99	4.00 (3.00–5.00)	4.16 ± 0.82	4.00 (3.00–5.00)	3.84 ± 0.73	KW = 8.502	0.014*	A < B
Q7	4.00 (2.00–5.00)	3.74 ± 0.82	4.00 (3.00–5.00)	4.29 ± 0.74	4.00 (3.00–5.00)	4.06 ± 0.63	KW = 7.830	0.020*	A < B
Q8	4.00 (2.00–5.00)	4.00 ± 0.86	4.00 (3.00–5.00)	4.16 ± 0.69	4.00 (2.00–5.00)	3.90 ± 0.83	KW = 1.301	0.522	–
Q9	4.00 (2.00–5.00)	4.23 ± 0.92	4.00 (3.00–5.00)	4.32 ± 0.65	4.00 (2.00–5.00)	3.68 ± 0.87	KW = 10.260	0.006*	C < A; C < B
Q10	4.00 (1.00–5.00)	3.71 ± 1.01	5.00 (3.00–5.00)	4.35 ± 0.75	4.00 (1.00–5.00)	3.84 ± 0.97	KW = 8.026	0.018*	A < B
Q11	4.00 (2.00–5.00)	4.06 ± 0.93	4.00 (1.00–5.00)	4.03 ± 0.91	4.00 (2.00–5.00)	3.77 ± 0.84	KW = 2.342	0.310	–
Q12	4.00 (2.00–5.00)	4.13 ± 0.96	3.00 (1.00–5.00)	3.35 ± 0.95	3.00 (2.00–5.00)	3.23 ± 0.92	KW = 15.455	< 0.001*	C < A; B < A
Q13	4.00 (1.00–5.00)	4.13 ± 0.96	4.00 (2.00–5.00)	4.00 ± 0.82	3.00 (1.00–5.00)	3.10 ± 1.25	KW = 13.903	0.001*	C < A; C < B
Q14	4.00 (2.00–5.00)	4.23 ± 0.88	4.00 (2.00–5.00)	4.03 ± 0.71	4.00 (2.00–5.00)	3.58 ± 0.67	KW = 11.904	0.003*	C < A
Q15	3.00 (2.00–5.00)	3.48 ± 0.72	5.00 (2.00–5.00)	4.35 ± 0.84	3.00 (2.00–5.00)	3.52 ± 0.72	KW = 20.813	< 0.001*	A < B; C < B
Q16	3.00 (2.00–5.00)	3.61 ± 0.80	4.00 (2.00–5.00)	4.19 ± 0.79	3.00 (2.00–5.00)	3.52 ± 1.00	KW = 10.468	0.005*	A < B; C < B
Q17	4.00 (2.00–5.00)	3.52 ± 0.89	4.00 (3.00–5.00)	4.26 ± 0.77	4.00 (2.00–5.00)	3.97 ± 0.84	KW = 10.301	0.006*	A < B
Q18	4.00 (2.00–5.00)	3.94 ± 0.85	4.00 (1.00–5.00)	3.77 ± 1.23	4.00 (2.00–5.00)	3.61 ± 1.02	KW = 1.352	0.509	–
Q19	4.00 (1.00–5.00)	3.55 ± 0.81	4.00 (2.00–5.00)	3.97 ± 0.84	4.00 (1.00–5.00)	4.03 ± 0.80	KW = 7.975	0.019*	A < C
Q20	5.00 (3.00–5.00)	4.29 ± 0.82	4.00 (2.00–5.00)	4.10 ± 0.87	4.00 (3.00–5.00)	4.06 ± 0.81	KW = 1.419	0.492	–

A, ChatGPT; B, Gemini; C, DeepSeek; SD, Standard Deviation; Min, Minimum; Max, Maximum; KW, Kruskal–Wallis test. *p < 0.05 indicates statistical significance. Post-hoc comparisons were performed using the Bonferroni correction to account for multiple testing.

**Fig. 1:**
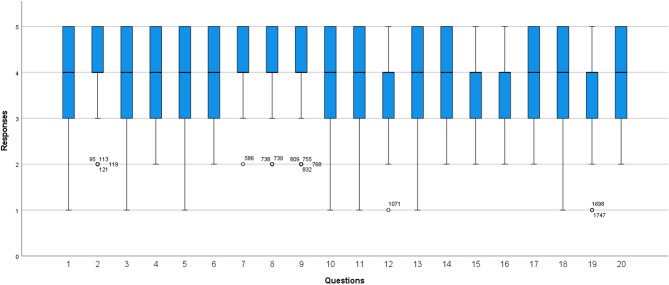
Box plot showing the distribution of mGQS for each question item.

Descriptive statistics for the LLM platforms’ performance regarding OCC information are presented in Fig. 1. Mean performance scores across all items ranged from 3.57 (Q12) to 4.15 (Q20), indicating a generally high quality in the platforms’ responses. Expert consensus was strongest for items Q2 and Q19, which exhibited the lowest variability (SD = 0.49). Conversely, the greatest divergence in expert evaluation was observed for item Q18, which showed the highest spread in scores (SD = 0.76).

**Fig. 2:**
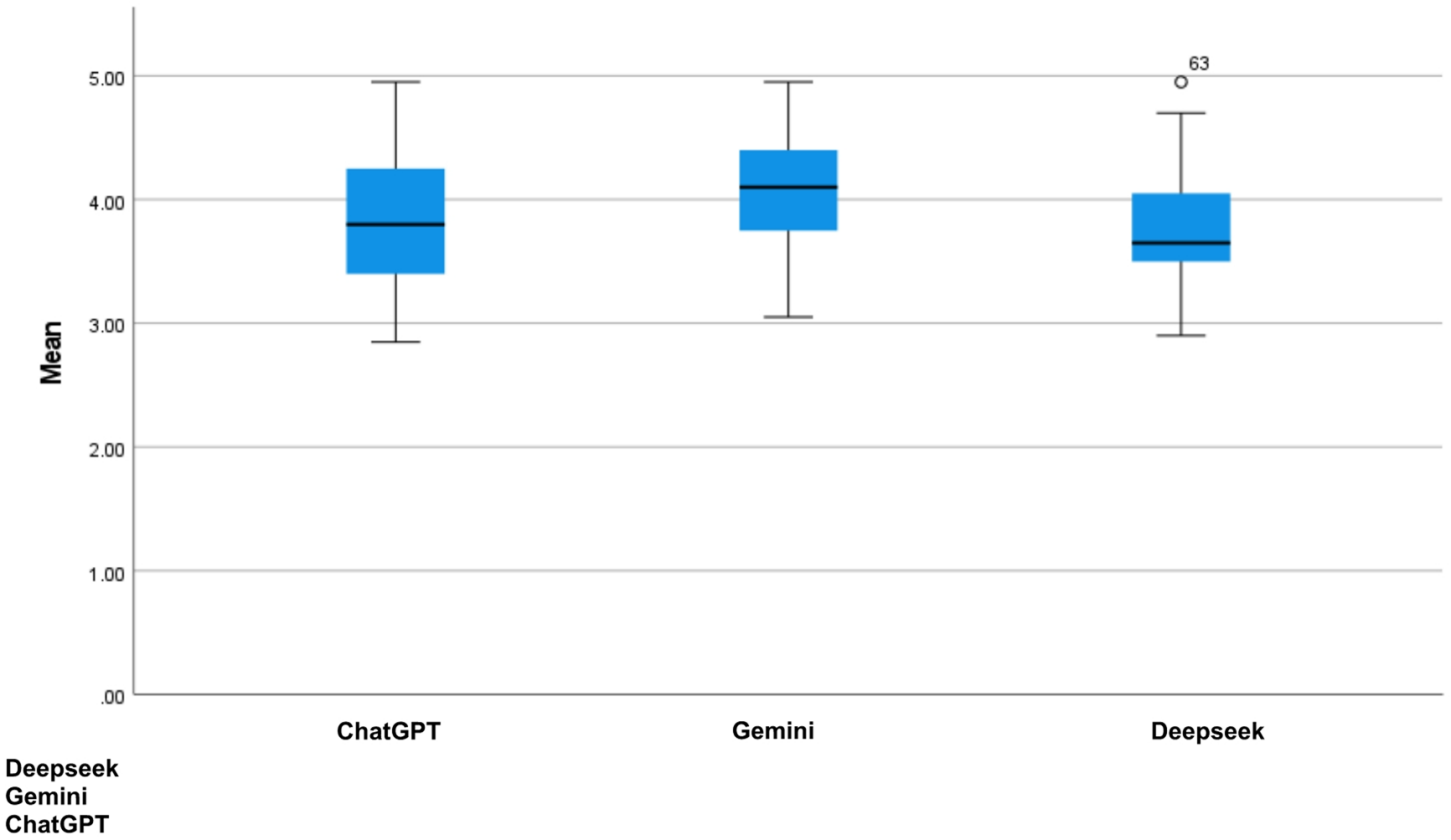
Box plot analysis illustrating the distribution of mGQS across different LLM platforms.

A statistically significant difference in overall evaluation scores was observed across the LLM platforms (*p* = 0.036). Further comparisons demonstrated that the difference originated from the DeepSeek–Gemini comparison, with DeepSeek showing significantly lower performance scores than Gemini (Fig. 2).

Significant differences in performance scores were identified among the LLM platforms in 15 of the 20 questions (Q1–Q3, Q5–Q7, Q9, Q10, Q12–Q17, and Q19; *p* < 0.05) (Table 3). Overall, the findings demonstrate heterogeneous performance in providing OCC information across most items. The most marked discrepancies were noted in items Q1, Q2, Q3, and Q5 (all *p* < 0.001), indicating that these topics contributed most to the observed performance variation between platforms.

Pairwise comparisons for the 15 items with significant differences are presented in Table 3. ChatGPT obtained significantly lower scores than Gemini and DeepSeek for items Q2 and Q3, but higher scores for items Q5 and Q12. Gemini achieved significantly higher scores than the other platforms for items Q15 and Q16. In contrast, DeepSeek scored significantly lower than the other two platforms for items Q9 and Q13. Item Q1 demonstrated a clear hierarchical pattern, with performance scores increasing from ChatGPT to Gemini and then to DeepSeek. Overall, these findings indicate complex and significant variability in LLM performance across most OCC-related content.

A significant difference among the specialty groups (OMFS, ENT, and MO) was observed only for question Q6 (*p* = 0.042; Table 4), with ENT specialists scoring lower than OMFS and MO specialists. No significant differences were found among the expert groups for the remaining 19 questions (Q1–Q5 and Q7–Q20, *p* > 0.05).

**Table 4:** Comparative analysis of responses according to specialty fields.

Question No	OMFS		ENT		MO		Test statistic	*p*	Post-hoc (Bonferroni)
	Median (Min–Max)	Mean ± SD	Median (Min–Max)	Mean ± SD	Median (Min–Max)	Mean ± SD			
Q1	4.00 (3.33–5.00)	4.03 ± 0.58	4.00 (3.33–4.33)	3.93 ± 0.38	4.00 (2.33–5.00)	3.73 ± 0.88	KW = 0.106	0.949	–
Q2	4.00 (3.67–5.00)	4.20 ± 0.39	4.00 (3.67–4.67)	4.03 ± 0.33	4.00 (3.00-.005)	4.00 ± 0.68	F = 0.469	0.631	–
Q3	4.00 (2.67–5.00)	3.93 ± 0.83	4.00 (3.33–4.33)	3.93 ± 0.31	4.00 (3.00–5.00)	4.06 ± 0.61	F = 0.184	0.833	–
Q4	4.17 (2.00–5.00)	3.93 ± 0.86	4.00 (3.00–4.33)	3.63 ± 0.55	4.00 (3.00–5.00)	3.97 ± 0.55	KW = 2.459	0.292	–
Q5	4.00 (3.33–5.00)	3.97 ± 0.58	3.67 (3.00–4.00)	3.53 ± 0.36	4.00 (2.67–5.00)	3.88 ± 0.86	F = 1.282	0.293	–
Q6	4.00 (3.00–5.00)	3.97 ± 0.53	3.33 (3.00–4.33)	3.43 ± 0.42	4.00 (3.00–5.00)	4.03 ± 0.67	F = 3.552	0.042*	B < A; B < C
Q7	4.17 (3.00–5.00)	4.07 ± 0.83	4.00 (3.33–4.33)	4.00 ± 0.31	4.00 (3.00–5.00)	4.03 ± 0.62	KW = 0.170	0.918	–
Q8	4.17 (3.00–5.00)	4.17 ± 0.77	3.67 (3.33–4.67)	3.87 ± 0.39	4.33 (3.00–5.00)	4.03 ± 0.62	F = 0.594	0.559	–
Q9	4.17 (2.67–5.00)	4.20 ± 0.65	4.00 (3.33–4.33)	4.03 ± 0.33	4.00 (3.00–5.00)	4.00 ± 0.76	KW = 1.097	0.578	–
Q10	4.17 (3.33–5.00)	4.20 ± 0.57	3.83 (2.33–5.00)	3.73 ± 0.73	4.00 (2.67–5.00)	3.97 ± 0.81	F = 1.064	0.358	–
Q11	3.83 (3.00–5.00)	3.93 ± 0.72	4.17 (2.67–5.00)	4.07 ± 0.77	4.00 (3.00–5.00)	3.88 ± 0.58	F = 0.203	0.817	–
Q12	4.00 (2.00–5.00)	3.77 ± 0.88	3.50 (2.00–4.00)	3.27 ± 0.60	3.67 (3.00–4.67)	3.67 ± 0.49	F = 1.562	0.227	–
Q13	4.17 (2.00–5.00)	3.87 ± 1.04	3.67 (2.67–4.00)	3.60 ± 0.47	4.00 (2.00–4.67)	3.76 ± 0.67	KW = 2.527	0.283	–
Q14	4.00 (3.00–4.67)	3.87 ± 0.57	4.17 (3.67–5.00)	4.10 ± 0.45	4.00 (2.67–4.67)	3.88 ± 0.54	KW = 0.827	0.661	–
Q15	3.83 (3.00–5.00)	3.83 ± 0.61	3.67 (3.00–4.00)	3.63 ± 0.29	4.00 (3.00–4.67)	3.88 ± 0.56	KW = 1.018	0.601	–
Q16	4.00 (2.00–5.00)	3.87 ± 0.92	3.50 (3.00–4.00)	3.60 ± 0.38	4.00 (3.33–4.33)	3.85 ± 0.31	KW = 2.277	0.320	–
Q17	4.17 (2.33–5.00)	4.00 ± 0.74	3.83 (3.00–4.67)	3.87 ± 0.50	3.67 (3.00–5.00)	3.88 ± 0.64	F = 0.137	0.872	–
Q18	4.17 (2.67–5.00)	4.00 ± 0.90	3.17 (2.67–4.00)	3.33 ± 0.54	4.00 (2.67–5.00)	3.97 ± 0.66	F = 2.809	0.077	–
Q19	4.17 (3.00–5.00)	4.13 ± 0.59	3.67 (2.67–4.00)	3.60 ± 0.44	4.00 (3.00–4.00)	3.82 ± 0.31	KW = 5.564	0.062	–
Q20	4.17 (3.00–5.00)	4.17 ± 0.63	3.67 (3.00–5.00)	4.00 ± 0.70	4.00 (3.00–5.00)	4.27 ± 0.70	F = 0.427	0.657	–

OMFS, Oral and Maxillofacial Surgery; ENT, Ear, Nose, and Throat; MO, Medical Oncology; SD, Standard Deviation; Min, Minimum; Max, Maximum; KW, Kruskal–Wallis test; F, ANOVA test statistic (Analysis of Variance). *p < 0.05 indicates statistical significance. Post-hoc comparisons were performed using the Bonferroni correction to account for multiple testing.

Evaluation by OMFS specialists demonstrated significant differences among the LLM platforms in 6 of the 20 questions (Q1, Q2, Q3, Q5, Q12, and Q16; Table 5). ChatGPT received the lowest scores for items Q1, Q2, and Q3, whereas its highest mean performance was observed for item Q12. DeepSeek scored significantly lower than the other platforms for items Q5 and Q16. No significant differences were identified among the platforms for the remaining 14 questions (*p* > 0.05).

**Table 5:** Comparison of question responses based on OMFS specialisation.

Question No	A		B		C		Test statistic	*p*	Post-hoc (Bonferroni)
	Median (Min–Max)	Mean ± SD	Median (Min–Max)	Mean ± SD	Median (Min–Max)	Mean ± SD			
Q1	3.00 (1.00–5.00)	3.30 ± 1.06	4.00 (3.00–5.00)	4.20 ± 0.63	5.00 (4.00–5.00)	4.60 ± 0.52	KW = 10.493	0.005*	A < B; A < C
Q2	3.00 (2.00–5.00)	3.40 ± 0.84	5.00 (4.00–5.00)	4.60 ± 0.52	5.00 (4.00–5.00)	4.60 ± 0.52	KW = 12.244	0.002*	A < B; A < C
Q3	3.00 (2.00–5.00)	3.30 ± 0.95	5.00 (3.00–5.00)	4.30 ± 0.95	4.50 (3.00–5.00)	4.20 ± 0.92	F = 3.441	0.047*	A < B; A < C
Q4	4.00 (2.00–5.00)	3.90 ± 1.10	4.00 (2.00–5.00)	4.00 ± 1.05	4.00 (2.00–5.00)	3.90 ± 0.99	KW = 0.079	0.961	–
Q5	4.50 (3.00–5.00)	4.40 ± 0.70	4.50 (3.00–5.00)	4.30 ± 0.82	3.00 (2.00–5.00)	3.20 ± 0.92	KW = 8.944	0.011*	C < B; C < A
Q6	4.00 (3.00–5.00)	3.70 ± 0.67	5.00 (3.00–5.00)	4.30 ± 0.95	4.00 (3.00–5.00)	3.90 ± 0.57	KW = 3.071	0.215	–
Q7	3.50 (3.00–5.00)	3.80 ± 0.92	5.00 (3.00–5.00)	4.40 ± 0.97	4.00 (3.00–5.00)	4.00 ± 0.82	KW = 2.373	0.305	–
Q8	4.50 (3.00–5.00)	4.20 ± 0.92	4.50 (3.00–5.00)	4.30 ± 0.82	4.00 (3.00–5.00)	4.00 ± 0.82	KW = 0.732	0.694	–
Q9	5.00 (3.00–5.00)	4.50 ± 0.71	4.50 (3.00–5.00)	4.40 ± 0.70	4.00 (2.00–5.00)	3.70 ± 0.82	KW = 5.904	0.052	–
Q10	4.00 (3.00–5.00)	4.00 ± 0.67	5.00 (3.00–5.00)	4.50 ± 0.71	4.00 (3.00–5.00)	4.10 ± 0.74	KW = 3.005	0.223	–
Q11	4.50 (3.00–5.00)	4.20 ± 0.92	4.00 (3.00–5.00)	4.00 ± 0.67	3.50 (2.00–5.00)	3.60 ± 0.97	KW = 2.228	0.328	–
Q12	5.00 (2.00–5.00)	4.50 ± 0.97	3.50 (2.00–5.00)	3.50 ± 0.85	3.00 (2.00–5.00)	3.30 ± 1.16	KW = 7.463	0.024*	C < A; B < A
Q13	5.00 (3.00–5.00)	4.30 ± 0.95	4.00 (2.00–5.00)	4.00 ± 1.05	3.50 (1.00–5.00)	3.30 ± 1.34	KW = 3.477	0.176	–
Q14	4.50 (3.00–5.00)	4.20 ± 0.92	4.00 (2.00–5.00)	4.00 ± 0.94	3.00 (3.00–4.00)	3.40 ± 0.52	KW = 5.120	0.077	–
Q15	4.00 (3.00–5.00)	3.80 ± 0.79	4.00 (3.00–5.00)	4.10 ± 0.88	3.50 (3.00–5.00)	3.60 ± 0.70	KW = 1.863	0.394	–
Q16	4.00 (2.00–5.00)	3.70 ± 0.95	5.00 (2.00–5.00)	4.50 ± 0.97	3.50 (2.00–5.00)	3.40 ± 1.17	KW = 6.143	0.046*	C < B
Q17	4.00 (2.00–5.00)	3.70 ± 0.82	4.50 (3.00–5.00)	4.20 ± 0.92	4.00 (2.00–5.00)	4.10 ± 0.99	KW = 1.980	0.372	–
Q18	4.00 (3.00–5.00)	4.20 ± 0.79	4.50 (2.00–5.00)	4.00 ± 1.15	4.00 (2.00–5.00)	3.80 ± 1.23	KW = 0.425	0.809	–
Q19	4.00 (3.00–5.00)	3.90 ± 0.74	4.00 (3.00–5.00)	4.20 ± 0.79	4.00 (3.00–5.00)	4.30 ± 0.67	KW = 1.607	0.448	–
Q20	4.50 (3.00–5.00)	4.20 ± 0.92	4.00 (3.00–5.00)	4.00 ± 0.67	4.00 (3.00–5.00)	4.30 ± 0.67	KW = 0.934	0.627	–

A, ChatGPT; B, Gemini; C, DeepSeek; SD, Standard Deviation; Min, Minimum; Max, Maximum; KW, Kruskal–Wallis test; F, ANOVA test statistic (Analysis of Variance). *p < 0.05 indicates statistical significance. Post-hoc comparisons were performed using the Bonferroni correction to account for multiple testing.

According to ENT specialists’ evaluations, significant inter-platform differences were observed in 13 of the 20 questions (Q1–Q3, Q5–Q7, Q9, and Q12–Q17; *p* < 0.05), indicating substantial variability in perceived platform performance. ChatGPT generally performed the worst, scoring significantly lower than Gemini and DeepSeek on items Q1, Q2, Q3, Q6, and Q7. DeepSeek also received significantly lower scores for items Q5, Q9, Q13, and Q14. In contrast, although Gemini was outperformed by ChatGPT on item Q12, it achieved significantly higher scores on items Q15, Q16, and Q17. No significant differences were found for the remaining seven questions (Q4, Q8, Q10, Q11, Q18, Q19, and Q20; *p* > 0.05; Table 6).

**Table 6:** Comparison of question responses based on ENT specialisation.

Question No	A		B		C		Test statistic	*p*	Post-hoc (Bonferroni)
	Median (Min–Max)	Mean ± SD	Median (Min–Max)	Mean ± SD	Median (Min–Max)	Mean ± SD			
Q1	3.00 (2.00–4.00)	3.10 ± 0.57	4.00 (3.00–5.00)	4.10 ± 0.74	5.00 (4.00–5.00)	4.60 ± 0.52	KW = 15.596	< 0.001*	A < B; A < C
Q2	3.00 (2.00–4.00)	3.20 ± 0.63	4.00 (4.00–5.00)	4.40 ± 0.52	4.50 (4.00–5.00)	4.50 ± 0.53	KW = 15.712	< 0.001*	A < B; A < C
Q3	3.00 (2.00–3.00)	2.90 ± 0.32	5.00 (4.00–5.00)	4.80 ± 0.42	4.00 (3.00–5.00)	4.10 ± 0.57	KW = 22.786	< 0.001*	A < B; A < C
Q4	3.50 (2.00–4.00)	3.30 ± 0.82	4.00 (3.00–5.00)	4.00 ± 0.94	4.00 (2.00–5.00)	3.60 ± 0.84	KW = 2.435	0.296	–
Q5	5.00 (4.00–5.00)	4.80 ± 0.42	4.00 (3.00–4.00)	3.60 ± 0.52	2.00 (1.00–4.00)	2.20 ± 0.92	KW = 22.485	< 0.001*	C < B; C < A; B < A
Q6	3.00 (2.00–4.00)	2.80 ± 0.79	4.00 (3.00–5.00)	4.20 ± 0.63	3.00 (3.00–4.00)	3.30 ± 0.48	KW = 13.528	0.001*	A < B; C < B
Q7	4.00 (3.00–4.00)	3.70 ± 0.48	4.00 (4.00–5.00)	4.30 ± 0.48	4.00 (3.00–5.00)	4.00 ± 0.47	KW = 6.525	0.038*	A < B
Q8	4.00 (3.00–5.00)	3.80 ± 0.63	4.00 (3.00–5.00)	4.00 ± 0.47	4.00 (3.00–5.00)	3.80 ± 0.63	KW = 0.908	0.635	–
Q9	4.50 (2.00–5.00)	4.20 ± 1.03	4.00 (4.00–5.00)	4.40 ± 0.52	3.50 (3.00–4.00)	3.50 ± 0.53	KW = 8.169	0.017*	C < A; C < B
Q10	3.00 (1.00–5.00)	3.30 ± 1.16	4.00 (3.00–5.00)	4.30 ± 0.67	4.00 (2.00–5.00)	3.60 ± 0.84	KW = 5.868	0.053	–
Q11	5.00 (3.00–5.00)	4.40 ± 0.84	4.00 (1.00–5.00)	4.00 ± 1.25	4.00 (2.00–5.00)	3.80 ± 0.79	KW = 2.918	0.232	–
Q12	4.00 (2.00–5.00)	4.10 ± 0.99	3.00 (1.00–4.00)	2.80 ± 0.92	3.00 (2.00–4.00)	2.90 ± 0.57	KW = 9.948	0.007*	C < A; B < A
Q13	4.50 (3.00–5.00)	4.40 ± 0.70	4.00 (3.00–5.00)	3.80 ± 0.63	3.00 (1.00–4.00)	2.60 ± 0.97	KW = 15.481	< 0.001*	C < A; C < B
Q14	5.00 (3.00–5.00)	4.50 ± 0.71	4.00 (4.00–5.00)	4.20 ± 0.42	3.50 (3.00–5.00)	3.60 ± 0.70	KW = 8.168	0.017*	C < A
Q15	3.00 (2.00–4.00)	3.10 ± 0.57	5.00 (4.00–5.00)	4.70 ± 0.48	3.00 (2.00–4.00)	3.10 ± 0.57	KW = 19.576	< 0.001*	A < B; C < B
Q16	3.00 (3.00–5.00)	3.20 ± 0.63	4.00 (3.00–5.00)	4.10 ± 0.57	3.00 (2.00–5.00)	3.50 ± 1.08	KW = 8.047	0.018*	C < B; A < B
Q17	3.50 (2.00–4.00)	3.30 ± 0.82	4.50 (4.00–5.00)	4.50 ± 0.53	4.00 (3.00–5.00)	3.80 ± 0.63	KW = 11.120	0.004*	A < B; C < B
Q18	3.50 (2.00–5.00)	3.50 ± 0.85	4.00 (1.00–5.00)	3.30 ± 1.25	3.00 (2.00–5.00)	3.20 ± 1.03	F = 0.209	0.813	–
Q19	4.00 (2.00–4.00)	3.50 ± 0.71	4.00 (2.00–4.00)	3.50 ± 0.85	4.00 (1.00–5.00)	3.80 ± 1.03	KW = 2.399	0.301	–
Q20	5.00 (3.00–5.00)	4.40 ± 0.84	3.50 (3.00–5.00)	3.80 ± 0.92	3.50 (3.00–5.00)	3.80 ± 0.92	KW = 2.900	0.235	–

A, ChatGPT; B, Gemini; C, DeepSeek; SD, Standard Deviation; Min, Minimum; Max, Maximum; KW, Kruskal–Wallis test; F, ANOVA test statistic (Analysis of Variance). *p < 0.05 indicates statistical significance. Post-hoc comparisons were performed using the Bonferroni correction to account for multiple testing.

Comparisons of the scores assigned by MO specialists revealed significant differences among the LLM platforms for questions Q1, Q2, and Q19 (*p* < 0.05; Table 7). For items Q1 and Q2, ChatGPT received significantly lower scores than DeepSeek, whereas for item Q19, ChatGPT scored significantly lower than Gemini. No significant differences were observed for the remaining questions (Q3–Q18 and Q20; *p* > 0.05).

**Table 7:** Comparison of question responses based on MO specialization.

Question No	A		B		C		Test statistic	*p*	Post-hoc (Bonferroni)
	Median (Min–Max)	Mean ± SD	Median (Min–Max)	Mean ± SD	Median (Min–Max)	Mean ± SD			
Q1	3.00 (1.00–5.00)	3.09 ± 1.14	4.00 (2.00–5.00)	3.64 ± 0.92	5.00 (3.00–5.00)	4.45 ± 0.82	KW = 9.098	0.011*	A < C
Q2	3.00 (2.00–5.00)	3.55 ± 1.13	4.00 (3.00–5.00)	3.91 ± 0.70	5.00 (4.00–5.00)	4.55 ± 0.52	KW = 6.475	0.039*	A < C
Q3	3.00 (1.00–5.00)	3.45 ± 1.44	5.00 (3.00–5.00)	4.45 ± 0.69	4.00 (3.00–5.00)	4.27 ± 0.65	KW = 3.395	0.183	–
Q4	5.00 (2.00–5.00)	4.18 ± 1.08	4.00 (3.00–5.00)	4.09 ± 0.83	3.00 (3.00–5.00)	3.64 ± 0.81	KW = 2.600	0.273	–
Q5	5.00 (2.00–5.00)	4.36 ± 1.03	3.00 (2.00–5.00)	3.45 ± 1.21	4.00 (1.00–5.00)	3.82 ± 1.25	KW = 3.570	0.168	–
Q6	4.00 (2.00–5.00)	3.82 ± 1.17	4.00 (3.00–5.00)	4.00 ± 0.89	4.00 (3.00–5.00)	4.27 ± 0.79	KW = 0.914	0.633	–
Q7	4.00 (2.00–5.00)	3.73 ± 1.01	4.00 (3.00–5.00)	4.18 ± 0.75	4.00 (3.00–5.00)	4.18 ± 0.60	KW = 1.773	0.412	–
Q8	4.00 (2.00–5.00)	4.00 ± 1.00	4.00 (3.00–5.00)	4.18 ± 0.75	4.00 (2.00–5.00)	3.91 ± 1.04	KW = 0.231	0.891	–
Q9	4.00 (2.00–5.00)	4.00 ± 1.00	4.00 (3.00–5.00)	4.18 ± 0.75	4.00 (2.00–5.00)	3.82 ± 1.17	KW = 0.395	0.821	–
Q10	4.00 (2.00–5.00)	3.82 ± 1.08	5.00 (3.00–5.00)	4.27 ± 0.90	4.00 (1.00–5.00)	3.82 ± 1.25	KW = 1.185	0.553	–
Q11	4.00 (2.00–5.00)	3.64 ± 0.92	4.00 (3.00–5.00)	4.09 ± 0.83	4.00 (3.00–5.00)	3.91 ± 0.83	KW = 1.364	0.506	–
Q12	4.00 (2.00–5.00)	3.82 ± 0.87	4.00 (2.00–5.00)	3.73 ± 0.90	3.00 (2.00–5.00)	3.45 ± 0.93	KW = 1.354	0.508	–
Q13	4.00 (1.00–5.00)	3.73 ± 1.10	4.00 (3.00–5.00)	4.18 ± 0.75	4.00 (1.00–5.00)	3.36 ± 1.36	KW = 2.117	0.347	–
Q14	4.00 (2.00–5.00)	4.00 ± 1.00	4.00 (3.00–5.00)	3.91 ± 0.70	4.00 (2.00–5.00)	3.73 ± 0.79	KW = 0.748	0.688	–
Q15	4.00 (2.00–4.00)	3.55 ± 0.69	5.00 (2.00–5.00)	4.27 ± 1.01	4.00 (3.00–5.00)	3.82 ± 0.75	KW = 4.925	0.085	–
Q16	4.00 (3.00–5.00)	3.91 ± 0.70	4.00 (3.00–5.00)	4.00 ± 0.77	4.00 (2.00–5.00)	3.64 ± 0.81	KW = 1.038	0.595	–
Q17	4.00 (2.00–5.00)	3.55 ± 1.04	4.00 (3.00–5.00)	4.09 ± 0.83	4.00 (3.00–5.00)	4.00 ± 0.89	KW = 1.756	0.416	–
Q18	4.00 (3.00–5.00)	4.09 ± 0.83	4.00 (1.00–5.00)	4.00 ± 1.26	4.00 (2.00–5.00)	3.82 ± 0.75	KW = 0.919	0.631	–
Q19	3.00 (1.00–4.00)	3.27 ± 0.90	4.00 (3.00–5.00)	4.18 ± 0.75	4.00 (3.00–5.00)	4.00 ± 0.63	KW = 6.785	0.034*	A < B
Q20	4.00 (3.00–5.00)	4.27 ± 0.79	5.00 (2.00–5.00)	4.45 ± 0.93	4.00 (3.00–5.00)	4.09 ± 0.83	KW = 1.665	0.435	–

A, ChatGPT; B, Gemini; C, DeepSeek; SD, Standard Deviation; Min, Minimum; Max, Maximum; KW, Kruskal–Wallis test; F, ANOVA test statistic (Analysis of Variance). *p < 0.05 indicates statistical significance. Post-hoc comparisons were performed using the Bonferroni correction to account for multiple testing.

## Discussion

The primary objective of this study was to evaluate experts’ perceptions of information quality provided by three prominent LLMs (ChatGPT-5.2, Gemini 3.1 Flash, and DeepSeek V4) regarding OCC. Our findings demonstrate that expert ratings for LLM outputs are highly heterogeneous and context-dependent. While Gemini received higher evaluation scores from specialists in complex domains such as survival outcomes and therapeutic principles, expert scores for ChatGPT were relatively lower in foundational categories, including symptomatology and risk factors. Conversely, DeepSeek’s scores were notably lower in topics concerning diagnostic modalities and distant metastasis. This intricate pattern underscores that favourable expert evaluation of an LLM’s response in one clinical area does not necessarily translate to similar levels of perceived quality across other clinical contexts.

Specialist evaluations showed high sensitivity to the specific clinical content of each query. Gemini was rated more favourably than both ChatGPT and DeepSeek in complex domains requiring contemporary evidence-based data, such as survival outcomes and therapeutic principles. In contrast, expert scores for ChatGPT were notably lower in foundational areas such as symptomatology, risk factors, and HPV-related associations, though it achieved higher scores on queries about regional clinico-pathological data, such as cervical metastasis rates. Conversely, DeepSeek received comparatively lower ratings in topics concerning diagnostic modalities, primary tumour localization, and distant metastasis.

The weak-to-moderate inter-expert consistency revealed by Fleiss’ Kappa coefficients suggests that even among domain experts, a unified consensus regarding the definition of an optimal OCC information response remains elusive. This variability indicates that specific mGQS parameters, such as the flow of information or the overall helpfulness of the response to the user, remain subject to individual and disciplinary interpretation. While the group-level analysis showed no significant differences in mean scores for 19 out of 20 items, this primarily indicates a lack of systematic divergence between specialties, despite the inherent subjectivity of the assessments. The low-to-moderate Kappa values may reflect differing clinical priorities across disciplines, such as surgical versus oncological perspectives, which are often inherent in multidisciplinary evaluations.

The mGQS is designed to evaluate the flow, readability, and perceived educational utility of information from a patient’s perspective, rather than serving as a direct audit of clinical correctness or adherence to medical guidelines^11^. Despite producing linguistically fluent and well-structured outputs, LLMs remain susceptible to 'hallucinations,' in which stylistic delivery may mask underlying factual inaccuracies^13^. Therefore, the high scores obtained in this study should be interpreted as evidence of superior information delivery and structural quality, rather than a definitive validation of scientific accuracy. Our findings reflect how well these models package medical information for laypeople, while verifying clinical factuality against established guidelines remains a distinct requirement for future research.

Consistent with previous evaluations of LLM proficiency in OCC, our results indicate that expert ratings vary by clinical category^3,14^. Yılmaz et al.^14^ reported that ChatGPT-4 Plus achieved its highest accuracy in recovery (72%) and treatment (62%) domains, whereas the diagnosis category exhibited the highest rate of inconsistencies. These observations parallel our findings, particularly regarding the variable specialist evaluations for ChatGPT in complex areas requiring advanced diagnostic synthesis and surgical reasoning. While Yılmaz et al. identified recovery as a strong point for ChatGPT, our multidisciplinary panel noted relatively lower scores on foundational topics such as symptomatology and risk factors, further reinforcing the notion that LLM outputs lack a uniform standard of reliability across all clinical subdomains.

Regarding topic-specific assessments, our findings vary from those reported by Çitir^3^, who observed that ChatGPT 3.5 was rated favourably in categories such as protection and symptoms. In contrast, our evaluation of a more recent iteration (ChatGPT-5.2) resulted in relatively lower specialist scores on foundational topics, including symptoms and risk factors. These discrepancies, alongside the comparative data for Gemini and DeepSeek, suggest that perceived information quality is influenced not only by the clinical subject matter but also by the evolution of underlying model types. While investigations limited to a single model may not fully capture these nuances, our multi-platform analysis highlights that specialist perceptions are shaped by both the chosen platform and the nature of the clinical topic.

Recent research in oral surgery comparing ChatGPT-4o, Claude-4, and Gemini-2.5 found no statistically significant difference between the models, even though Gemini achieved the highest mean diagnostic score^15^. Notably, the study reported that while these models could reach meaningful conclusions in moderately difficult cases, their performance markedly decreased in difficult scenarios^15^. These findings reinforce our results, suggesting that LLM superiority is not universal but varies by clinical context. Such variability underscores the critical importance of evaluating these tools within specific diagnostic categories and emphasizes that their supportive role remains most reliable when supervised by an expert, particularly in complex oncological or surgical cases.

Existing literature generally positions the ChatGPT-4 model as a prominent reference when evaluating the capabilities of various LLMs^16,17^. For instance, Rokhshad et al.^16^ compared ChatGPT-4, Bing, Bard, and Claude regarding OCC queries, noting that ChatGPT received favorable ratings in quality, empathy, and citation accuracy. In contrast, despite its strong standing in earlier reports, ChatGPT exhibited notable variation in specialist scores in our analysis, particularly trailing DeepSeek and Gemini on foundational topics.

Another study evaluating ChatGPT in Oral and Maxillofacial Surgery reported high general accuracy and quality ratings, yet identified a notable trend^17^. Specifically, specialist evaluations for high-difficulty questions were significantly lower than those for basic or intermediate queries. This suggests that the perceived reliability of LLM outputs may decrease in contexts requiring nuanced clinical reasoning or expert-level commentary. These observations align with the primary caution of our study: while LLMs may provide satisfactory general information, expert clinical verification remains essential for topics involving specialized medical knowledge.

Recent literature highlights the potential of LLMs as accessible tools for enhancing OCC awareness. A multidisciplinary study involving experts in behavioural sciences, oncology, and dentistry noted the high accessibility of these platforms while emphasizing the need for simplified terminology and improved visual presentation^18^. Consistent with these observations, our specialized panel (OMFS, ENT, and MO) assigned high content-quality scores to ChatGPT, Gemini, and DeepSeek, though the complexity of the generated content suggests a continued need to optimize information for patient use.

Literature across various medical subfields indicates that specialist evaluations of LLMs vary significantly by clinical domain. While Bashah et al.^19^ observed higher accuracy ratings for DeepSeek in salivary gland cancers, our study found a different trend where Gemini received more favourable overall scores. This suggests that different platforms may be perceived more effectively in distinct oncological niches. Similarly, research in dental trauma noted that ChatGPT was rated favourably for accuracy, whereas DeepSeek was preferred for its comprehensiveness^20^. Such discrepancies reinforce the view that no single platform holds universal superiority; rather, the perceived quality of LLM outputs appears to be highly domain-specific.

Despite methodological similarities, research by Yüceer Çetiner et al.^21^ on orthognathic surgery observed a different trend in platform evaluations compared to our findings. In their investigation, Gemini was noted as a consistently favoured platform across all evaluated parameters. In contrast, our study does not suggest the universal superiority of a single model; rather, our results indicate that specialist perceptions are multifaceted and vary by clinical topic. Furthermore, the observation by Yüceer Çetiner et al.^21^ that certain models may lack clinical safety despite high readability reinforces our assertion that linguistic quality is not a reliable indicator of medical accuracy.

The integration of LLMs into dental education shows varying degrees of success depending on the complexity of the assessment. Recent literature evaluating LLM performance on the Dentistry Specialization Entrance Examination (DUS) in Türkiye noted that ChatGPT achieved high accuracy, significantly exceeding the scores of Gemini and Copilot in textual categories^22^. However, a notable decline in ratings was observed for image-based inquiries, highlighting current limitations in multimodal processing within specialized clinical contexts. Our evaluation of ChatGPT and Gemini reflects this trend of favourable textual quality scores, while also illustrating the platforms’ rapidly evolving nature.

The perceived quality of LLM outputs in dentistry appears highly dependent on the clinical context and scenario complexity. Recent research utilizing dental examination questions noted that while platforms such as ChatGPT, DeepSeek, and Gemini received favourable ratings for textual content, their utility was limited in multimodal tasks, particularly with image-based queries^23^. Our findings, which utilized mGQS to evaluate OCC content, align with this trend of satisfactory textual quality in the latest-generation models. However, while some literature identifies salivary gland diseases as a less proficient domain, our specialized panel (OMFS, ENT, and MO) assigned the lowest scores to neck metastasis rates^23^. This specific discrepancy is clinically noteworthy, as the assessment of nodal involvement is essential for surgical staging and decision-making in OCC. Such variations highlight that while LLMs serve as valuable supplementary resources, they remain an adjunct to expert clinical judgment in high-stakes oncological contexts.

While applications of LLMs in OCC have primarily focused on clinician-oriented diagnostic support, such as the integration of algorithms with optical imaging for lesion detection, these technologies have evolved to serve a broader role in patient education^6,24,25^. Our study addresses a gap in this transition by evaluating the quality of information provided by modern chatbots. Although the literature suggests that LLMs can complement traditional methods in screening and risk modelling, significant hurdles, such as data heterogeneity and ethical requirements, persist^26,27^. By focusing on perceived information quality, our findings extend this discourse to include patient education workflows, illustrating how these tools may bridge the gap between clinical diagnostics and enhanced health literacy.

The high quality scores observed in our study align with emerging evidence regarding both the clinical potential and inherent limitations of LLMs. In a prospective comparison, Abou-Bakr et al.^28^ demonstrated that while the latest iterations of ChatGPT generated clinically useful differential diagnoses, they significantly lagged behind oral medicine experts in accuracy, particularly in complex inflammatory cases. This indicates that as diagnostic scenarios become more nuanced, the reliability of LLM outputs diminishes. Furthermore, a systematic review by Hassanein et al.^29^ involving over 1200 cases highlights that although LLMs excel in structuring differential reasoning and identifying critical indicators, they remain limited in tasks requiring fine morphological interpretation or severity grading. Crucially, the calibration of these models remains a concern; Hassanein et al.^30^ recently identified an optimistic bias in models like DeepSeek-V3, which frequently over-score incorrect or incomplete clinical responses. These collective findings confirm that while LLMs provide structurally sophisticated and seemingly expert-like information, they harbour critical reliability gaps in specialized oncological contexts. Therefore, LLM integration into clinical workflows must be limited to supervised settings in which the expert physician remains the final authority to ensure patient safety.

Beyond providing patient-oriented information, LLMs are emerging as transformative educational supports in dentistry. Recent evidence indicates that ChatGPT-4o-based, MeSH-guided personalized feedback significantly improves students’ radiographic diagnostic performance and learning satisfaction compared to conventional feedback methods^31^. Although the present study focuses on OCC information for patients, these educational findings suggest that LLMs can play a broader role in the dental field. Integrating these personalized LLM-based tools can create a more structured learning environment and help dental professionals better interpret complex clinical data.

While the LLMs in our study received high-quality scores, their human-like interaction models harbour a hidden risk for oncology patients. As emphasized by Lawson^4^, the high level of perceived realism and empathetic fluency provided by these tools can foster an unwarranted sense of trust. This creates a potential trust trap: patients may uncritically accept erroneous or hallucinatory information, misled by the chatbot’s persuasive tone. Consequently, the observed cross-platform variability underscores that LLM outputs must not be utilized in patient education without rigorous specialist filtering. To avoid the trust trap, the expert physician must remain the primary authority, ensuring that all AI-generated information is validated and supervised before clinical application.

A potential limitation of our study is the use of a single-response sampling strategy for each query. We acknowledge that LLMs are inherently non-deterministic and can produce variable outputs across different sessions due to their stochastic nature. However, our methodology was intentionally designed to simulate a real-world patient experience. In a typical scenario, a patient seeking health information interacts with an LLM through a single query and usually relies on the first comprehensive output provided, rather than performing multiple iterations or statistical sampling. By using default interface settings (including temperature) on premium accounts without prompt replication, we aimed to capture the primary response a user would encounter. While this approach reflects the immediate utility of LLMs for patients, future studies could employ repeated sampling methods to further explore the consistency and stability of these models’ performances.

While our study assessed perceived scientific quality, the formal validity of references and compliance with clinical guidelines were not evaluated. LLMs frequently exhibit academic hallucinations, with some studies reporting only 10% accuracy in provided citations^13^. Furthermore, evidence suggests an optimistic bias where models over-score incorrect clinical responses, necessitating expert verification before clinical use. Given the snapshot nature of our February 2026 data and the rapid evolution of LLMs, longitudinal studies are required to monitor the shifting reliability and performance levels of these iterative tools.

## Data Availability

The datasets generated and/or analyzed during the current study are available from the corresponding author on reasonable request.
